# Towards community-based nursing: Mothers’ experiences caring for their preterm infants in an informal settlement, Gauteng

**DOI:** 10.4102/hsag.v25i0.1437

**Published:** 2020-12-11

**Authors:** Alida S. du Plessis-Faurie, Marie Poggenpoel, Chris P.H. Myburgh, Wanda O. Jacobs

**Affiliations:** 1 Department of Nursing Sciences, Faculty of Health, University of Johannesburg, Johannesburg, South Africa

**Keywords:** Experiences, Mothers, Preterm infants, Informal settlement, Community clinic nurses

## Abstract

**Background:**

Pregnant women who experience preterm labour rush to public hospitals closest to the informal settlement in which they reside. Preterm infants are discharged when they reach a certain weight. Mothers take their preterm infants to their homes inside the informal settlements. Yet, preterm infants have special needs and require specific management. Research confirmed that nurses working in community clinics near informal settlements are unaware of the challenges faced by such mothers. Community nurses are at the heart of nursing, they work closest to the community and have a distinct opportunity to provide contextual, community-based care and support to these mothers, to promote good health and prevent diseases.

**Aim:**

This article aims to enhance community nurses’ insight about the mothers’ experiences in caring for their preterm infants post-hospitalisation.

**Setting:**

The study was conducted in an informal settlement in Midvaal, Gauteng.

**Methods:**

A qualitative, exploratory, descriptive and contextual research design was used. In-depth, phenomenological interviews were conducted with 10 purposefully sampled mothers to explore their experiences in caring for their preterm infants in an informal settlement. Data were analysed using Giorgi’s coding method. Ethical approval was received from the University of Johannesburg. Measures were applied to ensure trustworthiness.

**Results:**

Three themes emerged: mothers experienced intrapersonal responses, interpersonal responses and numerous physical challenges in taking care of their preterm infants.

**Conclusion:**

Study findings revealed that mothers experienced several responses in caring for their preterm infants. Sharing their experiences can enhance community clinic nurses’ insight to provide contextual health education.

## Introduction

Infants born prematurely in various public hospitals were discharged when they reached a certain weight and demonstrated stable clinical vital data, regardless of their gestational age (Ramdin et al. 2018; Williams 2019). Infants were discharged being premature into the care of their mothers who lived in informal settlements. Informal settlements are communities in South Africa holding sub-optimal resources (Chirau 2014; Naidoo, Piketh & Curtis 2014): most houses do not have running water inside and dwellings are generally noisy because of close proximity to each other (Midvaal Local Municipality 2017). The houses and situation in the informal settlement offered no complementary support to preterm infants discharged from hospital, despite being delicate and in need of special care by their mothers (Burnham, Feely & Sherrad 2013; Darmstadt, Shiffman & Lawn 2015; Sahni & Polin 2013). Preterm infants need specific care in meeting their thermoregulatory requirements to support and enhance their neuro-sensory system (Le Roux et al. 2015; Licata et al. 2014).

However, inadequate literature was available from Pub Med, Embase, CINAHL, UJoogle and Google Scholar databases about mothers’ experiences in having to care for their preterm infants whilst living in an informal settlement. The purpose of this study was therefore to develop an understanding of the mothers’ lived experiences in caring for their preterm infants in an informal settlement by exploring and describing their lived experiences of the phenomena.

## Research methods and design

### Study design

A qualitative, exploratory, descriptive phenomenological and contextual design was employed. The key concern for the researcher to use the qualitative research design was that the qualitative approach is related to the naturalistic inquiry (Lincoln & Guba 1985), which seeks to understand the world in which the participants live. The mothers’ experiences with regard to the care of their preterm infants whilst living in the informal settlement were explored and described in a systematic, interactive, subjective and holistic approach to give them meaning (Burns & Grove 2011). This included the description of the population and the sample, data collection, data analysis and measures to ensure trustworthiness. The conceptualisation process involved the exploration of literature to arrive at a meaningful interpretation and concluding statements, which formed the basis for recommendations.

### Setting

The study was conducted in an informal settlement located in the Southern region of Gauteng province (Midvaal Local Municipality 2015b). An estimated number of 2000 to 3500 families live in the informal settlement with an estimation of 7200 residents (Midvaal Local Municipality 2015; Ndlovu 2016; Yes Media 2012–2019). The population of the informal settlement included in the research expanded significantly in 10 years, increasing by 39% progressively. Nearly half of the potential labour force is unemployed and 50% of households live in poverty (Ndlovu 2016; Urban Genesis Management 2010).

### Population and sampling

This research focussed on the mothers’ lived experiences in caring for their preterm infants whilst living in an informal settlement. The researcher selected participants through a purposive sampling method by ensuring that they experienced the phenomenon in question (Denscombe 2007; Leavy 2017; Reiners 2012).

Ten adult mothers who lived in the informal settlement participated in the research. All the mothers gave birth to preterm infants, ranging from 30 to 36 weeks’ gestation. The preterm infants were admitted in various public hospitals’ neonatal wards or neonatal intensive care units for a minimum of 6 hours. The premature infants were discharged home, into the care of their mothers who lived in an informal settlement at the time of discharge.

During data collection, the participants’ children’s chronological ages varied between 8 weeks and 5 years old. Dains, Baumann and Scheibel (2016); Provenzi et al. (2016) and Sadock, Sadock and Ruiz (2015) suggest that at least 3 to 6 months should be allowed after the discharge of the preterm infant before interviews are undertaken, as mothers continue to find the unexpected birth of their preterm infant distressing. Two mothers of children aged 3 and 5 years old approached the researcher and requested to be included in the research, as their children were born premature. Interviewing the mothers after some time had passed ensured that they were settled in the idea that their infants were born prematurely, and they were able to reflect on their experiences in caring for their preterm infant in an informal settlement. The majority of mothers were able to converse in English. Those who communicated with difficulty in English but still wanted to participate in the study indicated their own interpreter who was able to translate during the interviews.

The gatekeeper, a female resident of the informal settlement, occupied the role as the chief community worker. Community members voted her into that leadership position long before this research commenced. She was undoubtedly respected as a leader, a confidant and at times a midwife. She knew the residents’ homes and circumstances and was able to identify mothers with preterm infants and informed them about the research without the researcher being present. This allowed the prospective participants to freely decide if they wanted to take part in the study. Only on confirmation of participating in the research did the gatekeeper accompany the researcher to meet the participants. The researcher’s first visit to the participants involved the gatekeeper who introduced the researcher to the mothers. Thereafter, the researcher proceeded to casually converse with the potential participants in the presence of the gatekeeper. Once the participant verbally confirmed willingness to participate in the study to the researcher, did the researcher arrange a follow-up date and time for the interview. Informed written consent for participation, audio-recording of interviews and taking of photographs of the environment was obtained during the subsequent meeting prior to the commencement of the interview. The gatekeeper remained present during some of the interviews when the participants indicated that they wanted her to be at hand for reassurance or possible interpretation. If the gatekeeper established the participant’s ease with the researcher, she would leave the interview so to continue with her own work in the community and allow participant privacy.

### Data collection

Data were collected by the first author from December 2016 to July 2017 until data saturation occurred. Phenomenological interviews occurred in English, inside the mothers’ homes, during the daytime, on a date and time that suited the participants best. The researcher initiated the interview with one central research question: ‘*How is it caring for your baby at home?*’ In-depth interview methods were used in an interactive and conversant manner to ensure rich data to gain understanding of the mothers’ lived experiences. Mothers who demonstrated difficulty in conversing in English were given an opportunity to select their own interpreter who translated stories between the researcher and the mothers. Interpreters also signed confidentiality agreements. Sometimes conversation occurred in the mothers’ language between herself and the interpreter, and the researcher allowed the conversation to ensue as it was audio recorded. At such times, the researcher resumed field notes whilst observing the participants’ body language and external circumstances which initiated them to converse in their mother tongue with each other. Interviews lasted 40 to 90 min, depending on the mothers’ need for a translator or ability to communicate comfortably in English. A translator who knew the mothers’ languages assisted to transcribe and translate the data which added value to the research data.

### Data analysis

Data were analysed using Giorgi’s phenomenological descriptive data analysis method (Giorgi 1985). The researcher transcribed the first interview as soon as possible after meeting with the mother to engage with the content. The rest of the interviews were transcribed by an accredited transcriber into English and the transcriber signed a confidentiality agreement. Field notes were recorded by the researcher and arranged per recording to consolidate it with the final transcribed material. The non-English recordings were transcribed in the various African languages and translated to English by a translator, who also signed a confidentiality agreement. The researcher read through all the transcriptions to add the specific field notes to each transcript and develop a sense of the whole representation. It was vital to do so, as phenomenology is holistic and concentrates originally on the ‘gestalt’, which means ‘the whole’. Once the whole depiction of the entire transcript was grasped, the researcher established the elements of the description, made ‘meaning units’ and discerned between the ‘meaning units’. The elements were relevant and centred on the phenomenon under study. The researcher ensured that the elements were not theory-loaded, but that it represented the language applied in everyday life. When the ‘meaning units’ were clarified, the researcher actively converted the initial data and articulated the perception confined in it. The researcher continued to emphasise collective themes, which were demonstrated in the participants’ quotations.

The researcher made the abstract data meaningful by describing the themes using examples of the mothers’ lived experiences, as guided by Giorgi (1985). The researcher described situations of the mothers’ lived experiences to demonstrate the themes. Transformed ‘meaning units’ were combined into constant statements about the mothers’ experiences using their statements which demonstrated the foundation of their experiences. Even though the researcher exposed the core of the mothers’ experiences as presented in the themes, the researcher referred to the phenomenon whilst focusing on the mothers’ stories. The researcher searched for the overall core of their experiences. An independent coder, being an expert in qualitative research, also analysed the data to safeguard objectivity of the researcher and decrease bias. Once both the researcher and independent coder reached consensus about the findings, the data were recontextualised into literature.

### Trustworthiness

Principles of trustworthiness according to Lincoln and Guba (1985) were followed. The research implemented in-depth, individual interviews, field notes and observational notes to ensure credibility. The researcher used deep-rooted research methods and described the methods with satisfactory detail in order for the research to be replicated. Various literature sources such as articles, Internet searches and books were used to enhance credibility of the research findings. Bracketing was achieved by identifying and acknowledging preconceived beliefs and opinions about the phenomenon under study by the researcher. A reflexive diary was maintained by the researcher in an effort to bracket by noting things that could be taken for granted. Personal values were clarified; areas of bias and feelings that would indicate a lack of neutrality or possible conflict were recognised. The researcher engaged in an unrehearsed relationship with the gatekeeper and some of the mothers, individualising the quality of the relationship to each mother. Although the relationship needed to be relaxed, the principle of mutual respect was upheld at all times.

Transferability was reached through purposive sampling of participants. Dependability was achieved by the code–recode method of analysis, where data were coded over an extended period of time to ensure consistency of the coding strategy. Personal notes and field notes were kept by the researcher. Confirmability was enriched by a dense description of results with direct quotations from participants as well as a confirmability audit, where the study supervisors audited the research project.

### Ethical considerations

Informed written consent was obtained from each mother before every interview commenced. With the mothers’ permission, each interview was audio-recorded. Mothers were informed that they could freely withdraw from the research at any time without consequences. The researcher numbered each interview transcript to ensure anonymity and confidentiality. Research data and the master list of the mothers’ names and matching numbers were kept in a locked cupboard, as well as a password encrypted electronic file that could only be accessed by the researcher and the study supervisors. Information gathered about the mothers was kept in a manner that did not link them to the specific information. The principle of justice concerning fair treatment and the right to privacy and anonymity in maintaining the researcher–participant relationship was observed. Ethical approval to conduct the study was obtained before the study commenced from the University of Johannesburg’s Faculty of Health Sciences Research Ethics Committee (reference number REC-01-152-2016).

## Findings of the study

### Demographic profile of the participants

The 10 participants in this study were mothers ranging between 21 and 35 years old. All of them gave birth to preterm infants between 30 and 36 weeks’ gestation. At the time of the research, the mothers’ children were aged 8 weeks to 5 years old. Two mothers were employed full-time, one being a community worker and the other one a factory worker. Three mothers were employed part-time, working as domestic workers. One mother was self-employed, managing her own shop selling vegetables to the local community. Four mothers were unemployed. Three mothers attended school until the secondary school level. Seven mothers attended school only up to the primary school level. Nine mothers lived in *mkhukhus* [corrugated iron houses]. One mother lived in a brick house. All mothers could speak English, but four participants requested the assistance of an interpreter whom they had selected themselves, as they realised during the interview that they were limited to converse in English comfortably.

### Themes

Three themes emerged from the data, which were identified as the mothers experiencing intrapersonal responses, interpersonal responses, combined with facing multiple physical challenges in their poor living environment. The researchers provide quotations of the mothers as example of all participants’ experiences.

Table 1 presents the three themes which emerged from the data.

**TABLE 1:** Summary of themes demonstrating the mothers caring for their preterm infants in an informal settlement.

Theme 1	The mothers experienced intrapersonal responses whilst taking care of their preterm infants in an informal settlement
Theme 2	The mothers experienced interpersonal responses whilst taking care of their preterm infants in an informal settlement
Theme 3	The mothers experienced a multitude of physical challenges in poor living conditions whilst taking care of their preterm infants in an informal settlement

*Source:* Adapted from Du Plessis-Faurie, A.S., 2019, ‘A model for nurses to facilitate mothers’ caring of their preterm infants in an informal settlement, Gauteng’, PhD thesis, University of Johannesburg

#### Theme 1: The mothers experienced intrapersonal responses whilst taking care of their preterm infants in an informal settlement

The first theme identified by the researchers was the mothers’ experiences demonstrated as intrapersonal responses in taking care of their preterm infants at home in an informal settlement. The conversations between the researcher and mothers emphasised their lived experiences in caring for their preterm infants whilst living in an informal settlement. In sharing their stories, the researcher developed an understanding of the mothers’ deeper feelings towards their preterm infants and the way they had to care for them. The mothers’ inner conflicts were identified in their expressions of distress at having to take care of an infant, particularly one born prematurely, being very small and fragile.

Some mothers experienced negative feelings in caring for a preterm infant:

‘I was angry because sometimes I think it’s going to fall, yes, yoh [*in dread*] because she was too little. Okay, I was angry because she got her birth early yes.’ (Participant 4, 30 years, lives in a brick house)

Some mothers’ stories demonstrated that they were scared of the preterm infants and they did not know how to deal with them:

‘Okay, she says she was very heartbroken. Because she think the baby will die. He was too small.’ (Interpreter for Participant 7, 21 years, lives in a *mkhukhu*)

‘I don’t understand the premature babies.’ (Participant 1, 26 years, lives in a *mkhukhu*)

Some mothers experienced negative encounters in caring for their preterm infants (Moghaddam Tabrizi et al. 2017; Van Schalkwyk et al. 2020) as manifested in this research. Reasons for their negative experiences included their awareness of the risk of their infants’ prematurity, concern about the early discharge of the preterm infant and the challenges that they experienced in caring for their preterm infants (Shiltz et al. 2014; Vonderheid et al. 2016). Shiltz et al. (2014), Van Schalkwyk et al. (2020) and Vonderheid et al. (2016) confirmed that preterm infants’ health problems and mothers seeking health care, compelled by their distress, are the greatest during the first few months after original hospital discharge.

#### Theme 2: The mothers experienced interpersonal responses whilst taking care of their preterm infants in an informal settlement

The subsequent theme was the mothers’ interpersonal experiences in taking care of their preterm infants at home in an informal settlement.

The stories shared with the researcher demonstrated interpersonal responses from their life partners, their family members and close friends, as well as their neighbours. Sharing their interpersonal responses helped the researcher to understand the mothers’ experiences whilst taking care of their preterm infants.

Life partners supported the mothers by paying bills, buying food or seeing to the items which the infants needed, specific to preterm infants. In the following quotation, the participant referred to her partner being able to support her when he received an income. Her partner had a part-time employment and she perceived his occasional financial support as honourable support, positively influencing their relationship.

‘But my boyfriend, he did help. Sometimes he does help me. He does help me. Took him, take him and do kangaroo.’ (Participant 1, 26 years, lives in a *mkhukhu*)

These findings were echoed in the study of Lydon et al. (2018) confirming that fathers had both positive and negative responses in caring for their preterm infants. Fathers experienced negative responses, as reflected in the stories where life partners were completely absent, sometimes aggressive towards the mothers and provided no financial support in caring for the preterm infants:

‘She said you don’t have food in your house, you think: Sometimes one week, no food. Your boyfriend he go to tavern. He shout. You fight. Every day. Every day your husband, if he has have money, he goes to the tavern.’ (Interpreter for Participant 10, 28 years, lives in a *mkhukhu*)

Adama, Sundin and Bayes (2017) confirmed in their research that some fathers were non-supportive. Gibbs, Sikweyiya and Jewkes (2014) accentuated that some men perceived respect through exercising control over their life partners by withholding finances, through violence against their life partners, by pursuing multiple sexual partners and displaying violence against other men in the same environment where the mothers and infants lived, putting their lives in danger. These findings confirmed the stories which the mothers in this study shared with the researcher in cases where their life partners did not support them in the care of their child. Finances played a vital role in caring for the preterm infants, as money was required to purchase wood or paraffin to provide warmth in the house, boil water to sterilise bottles, prepare feeds and bath the infants. Money was vital for the mothers to care for their preterm infants as they needed to purchase particular nappies, formula milk, blankets and clothes, or taking their preterm infants to the clinic for follow-up care. Boutain, Foreman and Hitti (2017) confirmed that mothers perceived their relationship with their life partners as challenging at the time of caring for their preterm infants, which was echoed by some of the mothers’ stories in this study.

#### Theme 3: The mothers experienced a multitude of physical challenges in poor living conditions whilst taking care of their preterm infants in an informal settlement

The researcher discovered a multitude of facts about the physical living conditions and the lived experiences of mothers caring for their preterm infants in an informal settlement. First, the mothers experienced frustration and despair having to deal with their physical environment. They suffered anxiety about their financial circumstances, causing some to start working very soon after birth and having to find someone willing to look after their preterm infant. As part of their poor living conditions, the mothers experienced extreme challenges in practising good hygiene, to the extent that they sometimes blatantly gave up trying to maintain good hygienic practices.

The participant shared how important the use of the primus stove was to provide in her baby’s nutritional needs:

‘“*Phantsi-phezulu*,” up and down, which means up and down and down in here. And then, I bought sterilizing fluid, and then I was boiling water, with a primastove. And then after boiling water, I took that water and pour it in a flask, so that water will always be warm, ne? Because I did not want to always boil water when I do his bottle. I said: “Joh [*awareness*], it is a lot of work. And it is winter. And it is cold eyh [*incredible*]. I won’t do that”’. (Participant 1, 26 years, lives in a *mkhukhu*)

‘I don’t like the *mkukhu*. Sometimes it, the rain is coming and the *mkukhu* it is not all right. Sometimes the rain is coming at night. You are not relaxed because of *mkukhu*. The wind is coming too much, you know we are not relaxed in *mkukhu*. Sometimes we don’t have blankets ma’am. Sometimes we use a primer to, paraffin, we are not relaxed. I am not happy ma’am but it is where I must stay.’ (Participant 8, 29 years, lives in a *mkhukhu*)

However, it is clear that some participants lived in such hopelessness that they did not even realise their preterm infants’ special needs. They experienced a comprehensive feeling of distraught because of the lack of finances and having to survive:

‘It’s not easy … Because if I was working it was better. Sometimes we sleep without eating.’ (Participant 3, 23 years, lives in a *mkhukhu*)

Some mothers made an effort to implement hand hygiene before and after touching their preterm infants, before cooking and after toilet use. But participants surrendered to the effort because it was hard work to collect water, store and separate the water, and still practice hand hygiene:

‘Where do you wash your hands [*cynical laugh*]? This is hard where do you wash your hands after going to the toilet or doing anything? Where do you wash your hands when you come back?’ (Interpreter translating for Participant 5, 32 years, lives in a *mkhukhu*)

James (2015) investigated residents’ perceptions of environmental health in the informal settlement in which they lived: poor living circumstances and conditions, combined with a lack of basic services were the main characteristics of informal settlements. Participants experienced an absence or shortage of basic services such as sanitation, water and electricity, which compromised the environmental health of the area. Vonderheid et al. (2016) confirmed that having a preterm infant meant substantially more expenses for the parents during the initial hospitalisation and after discharge. This statement was reiterated by research conducted by Petrou (2019). Finances were definitely a challenge when caring for a preterm infant. Having a single income was difficult. Participants with life partners who provided financially in caring for the preterm infants still mentioned the money being insufficient, as the preterm birth of the infant added to the costs of daily living (Boutain et al. 2017).

Findings in the mentioned literature, compared with the findings of the study undertaken in the informal settlement regarding financial constraints, confirmed additional financial expenses in caring for a preterm infant, and the urgency to find a job to meet the financial responsibilities.

Oldewage-Theron and Slabbert (2008, 2010) stated that their survey found a 91% degree of unemployment in the same area where the informal settlement included in this study was located. This resonated with mothers sharing their life stories where they ‘went to bed without eating’, had to return back to work sooner than planned and were unable to afford basic items needed to care for their preterm infants whilst living inside the informal settlement.

## Discussion

This study materialised within the paradigm of postmodern social constructivism utilising a theory-generating, qualitative, exploratory, descriptive and contextual research design. Mothers taking care of their preterm infants in an informal settlement became overwhelmed with the responsibility of caring for their preterm infants under trying circumstances. They became despondent in making an effort to care for their preterm infant in an optimal manner. They were overwhelmed with negative responses like concern, stress and fear related to their preterm infants. Others were concerned about their loved ones being drawn into the care of the preterm infant and the added responsibilities they carried to continue with the normal daily living challenges and considering the special efforts to be made in caring for the preterm infant in the informal settlement. Some mothers had little or no support from their life partners, family or friends in caring for their preterm infants. They had to carry the brunt of the responsibilities in taking care of their preterm infants. All the mothers experienced multiple physical challenges in taking care of their preterm infants in circumstances completely unconducive for the health of preterm infants.

Being unable to change the social and physical circumstances inside the informal settlement, all the above-mentioned experiences left the mothers worn-out and disempowered whilst caring for their preterm infants, living in the informal settlement.

Research findings were verified and substantiated with literature in order to place the identified concepts in relation to the research results within this context. No literature was found confirming mothers’ experiences in caring for their preterm infants in an informal settlement after discharge from hospital. Literature which detailed mothers’ experiences in caring for their preterm infants during hospitalisation and after discharge in non-defined community contexts, were used to identify and support central concepts from the results. Literature control provided re-contextualisation of findings and resulted in current, existing nursing and other related theoretical literature.

The researcher believes that the condition of the mothers’ living circumstances would not improve in the informal settlement. It is expected that there will always be infants who are born prematurely. A multitude of literature based on research exists about mothers’ experiences in giving birth to preterm infants, caring for them in neonatal intensive care units (NICUs) and taking them home. Literature also exists about the circumstances inside informal settlements. However, to date, no research has explored the mothers’ lived experiences in having to care for a preterm infant in an informal settlement in Gauteng. Therefore, the purpose of this article is to demonstrate the experiences of mothers caring for their preterm infants in an informal settlement, in order for nurses working in the community clinics to provide contextual and individualised health education.

## Strengths and limitations of the study

This study delivered an original contribution to the body of knowledge with respect to nursing in South Africa, especially towards mother and child health and specifically on preterm infants in the community. Being knowledgeable about the mothers’ experiences in caring for their preterm infants in an informal settlement, is significant as it contributes to the field of nursing research and nursing practice into the aspects of community health science, combined with aspects of midwifery nursing science and neonatal nursing science. Community health science combined with neonatal nursing science has not been addressed in South Africa and is therefore very relevant for the nursing practice.

## Recommendations

Nurses working in community clinics serving an informal settlement need to comprehend what transpires when mothers attempt to integrate into the community with their preterm infant after discharge from hospital, as reiterated in the findings of the research by Gullino et al. (2017). They should be assisted by community health nurses to transform their hospital motherhood experiences into their own social context as it will support their caring and also enhance their preterm infants’ wellbeing and development. The experiences of mothers caring for their preterm infants inside a hospital, with all the resources at hand — nurses’ advice, water, electricity and warmth — were in stark contrast to the situation inside the informal settlement where trained nurses, familiar with preterm infants, were not immediately available, and every life-sustaining resource like water, electricity and warmth had to be collected with great effort.

Community health care is central to uplifting and supporting the South African health system (Rispel 2016). It is therefore highly recommended that knowledge about the mothers’ experiences be considered by nurses working in the community clinics which serve informal settlements. Nurses working in community clinics dealing with mothers caring for their preterm infants in informal settlements can greatly benefit by applying this knowledge into their practice.

Programmes are in place for the control of HIV and/or AIDS, the management of non-communicable diseases and communicable diseases, maternal health programmes focusing on antenatal care, postnatal care, and caring for the full-term infant (Machingaidze et al. 2017). However, no information exists on the management of preterm infants in the community, particularly in the informal settlement. No guidelines or programmes are available for nurses working in community clinics to provide contextual and individualised health education for mothers caring for particularly preterm infants inside an informal settlement.

## Conclusion

This study identified that the mothers experienced various responses in caring for their preterm infants in the informal settlement. The Department of Health (n.d.) provides guidelines in the Integrated Clinical Services Management for clinic nurses about newborn care in the community. But, the guideline does not provide management principles for preterm infants in the clinics, more so, no advice about the care of preterm infants living inside an informal settlement. The study of Currie et al. (2018) confirmed that nurses working in the community clinics need to know more about preterm infants in order to render quality care and support for families once their preterm infants are discharged. Being knowledgeable about the mothers’ real-life experiences can enhance the efficiency of nurses working in the community clinics. This article aids nurses in contextualising and individualising their health education to such mothers, particularly towards caring for their preterm infants in the informal settlement.
